# Implementation of Free Text Format Nursing Diagnoses at a University Hospital's Medical Department. Exploring Nurses' and Nursing Students' Experiences on Use and Usefulness. A Qualitative Study

**DOI:** 10.1155/2015/179275

**Published:** 2015-05-13

**Authors:** Sigrun Aasen Frigstad, Torunn Hatlen Nøst, Beate André

**Affiliations:** ^1^Faculty of Nursing, Sør-Trøndelag University College (HiST), Postboks 2320, 7004 Trondheim, Norway; ^2^St. Olav's Hospital, Trondheim University Hospital, Postboks 3250, 7006 Trondheim, Norway

## Abstract

*Background*. Nursing documentation has long traditions and represents core element of nursing, but the documentation is often criticized of being incomplete. Nursing diagnoses are an important research topic in nursing in terms of quality of nursing assessment, interventions, and outcome in addition to facilitating communication and continuity. *Aim*. The aim of this study was to explore the nurses' and nursing students' experiences after implementing free text format nursing diagnoses in a medical department. *Method*. The study design included educational intervention of free text nursing diagnoses. Data was collected through five focus group interviews with 18 nurses and 6 students as informants. The data was analyzed using qualitative content analysis. *Results*. The informants describe positive experiences concerning free text format nursing diagnoses' use and usefulness; it promotes reflection and discussion and is described as a useful tool in the diagnostic process, though it was challenging to find the diagnosis' appropriate formulation. *Conclusion*. Our findings indicate a valid usability of free text format nursing diagnoses as it promotes the diagnostic process. The use seems to enhance critical thinking and may serve as valuable preparation towards an implementation of standardized nursing diagnoses. Use and support of key personnel seem valuable in an implementation process.

## 1. Introduction

This paper reports nurses' and nursing students' perceptions of use and usefulness after implementation of free text nursing diagnoses (FTF-ND) with a Problem-Etiology-Symptom structure (PES). It represents the qualitative part of a study addressing three different yet related aims. The first aim was to investigate the implications of implementing PES-structured FTF-ND at a medical department by means of an educational intervention and the effect of this on nursing documentation in electronic health records (EHR). The second aim was to explore nurses' and nursing students' perceptions of clinical use and usefulness. The third aim was to study the impacts of the implementation of nursing diagnoses on work culture. A research collaboration was established between a hospital and a university college to study these aims. The quantitative part of the study measured the effect of implementing nursing diagnoses by using N Catch (Norwegian Catch), an audit instrument of nursing documentation. This instrument is translated and adjusted to a Norwegian setting, based on the audit instrument D-Catch [1, 2] in order to assess quality and quantity of nursing documentation in EHR before and after the educational intervention (work in progress). The effect of implementation on work culture was measured by the SPGR method (Systematizing Person-Group Relations) which seeks to identify aspects dominating the particular work culture [3, 4]. This paper presents the results of the study's second aim.

### 1.1. Background

Nursing documentation is considered a core element of nursing with a specific purpose of securing well planned, implemented, evaluated, and documented patient care as well as promoting communication among the caregivers. An additional profit of quality nursing documentation is the opportunity in facilitating continuity, individualized care, and patient safety [5–7]. Nurses have a long tradition in documenting patient observations and assessments. It has been mandatory by law for nurses to describe planned, given, and evaluated health care in patient records in Norway since 2001 [8]. Despite the recognition and importance, in addition to implementation of EHR over the last decade, national research and international research reveal obvious challenges related to nursing documentation's accuracy, content. and completeness. It still uncovers nursing records as problematical [9–14].

The nursing process model constitutes the acknowledged structure of nursing records and nurse care plans and furthermore includes the theoretical foundation enabling completeness and accuracy in documentation [13, 15–18]. The different phases of the nursing process represent the systematics supporting the nurse work process, constituting the five steps of assessment/data collection, nurse diagnosis/need identification, planning/identifying goals and interventions, implementation, and evaluation/identifying outcome. The nursing diagnosis is considered a core element in the nursing process as it guides and directs nursing care in addition to promoting the documentation process itself [6, 13, 16, 19, 20]. PES is an international, recognized structure of a nursing diagnosis consisting of the elements problem/health need, etiology, and symptom, independent of any classification system. Its purpose is to grasp the compound essence of a health need requesting nurse care [2, 21, 22]. Use of the term* P(R)ES structure* may be seen in some literature, emphasizing the *R* as either a risk or resource. The PES structure is applicable for free text format as well as classification use [6, 23]. Focus on utilization and development of nursing diagnoses is apparent within nursing research, related to the improvement of quality in nursing assessments, interventions, and outcome. Enhancement of communication and continuity among health workers is revealed as an additional profit. Nurses describe nursing diagnoses as a tool of grasping patient complexity [24, 25]. The concept of critical thinking is closely connected to clinical use of nursing diagnoses but still lacks a joint nursing consensus. A recent systematic review [26] points out common significant characteristics in the concept, such as cognitive abilities of interpretation, exploration, evaluation, analysis, decision making, and self-regulation. Critical thinking is essential in order to assess patient situations and to undertake well-grounded clinical decisions [26–28]. Critical thinking can be identified through terms of cognitive skills and habits of mind, as interpersonal, technical, and intellectual competencies. The ability of critical thinking increases individualized care [27]. Use of critical thinking enables the nurse to increase accuracy of nursing diagnoses. It is described as a complex thinking process demanding different skills depending on the specific patient situation [19, 27, 29, 30].

Although nursing educational programs emphasize curriculum within the nursing process and nursing diagnoses as significant elements in developing skills of critical thinking, it appears problematic for students to apply this in practice [18, 31]. Challenges seen in nursing education disclose a need to uphold a distinct focus on critical thinking due to a competing instrumental educational ideology [32]. Nursing students emphasize technical skills and display an instrumental knowledge approach despite the fact that education attempts to increase the emphasis on critical thinking. This is stated as an educational challenge in Norway [33]. Clinical assignments provide opportunities in developing nursing students' critical thinking, often structured within the nursing process framework [18]. Students indicate strengthened confidence of thinking skills when challenged to reflect on specific patient care related questions as opposed to traditional care plan assignments. This is in accordance with current research indicating how students' ability of critical thinking differs between various learning styles [34, 35]. Educational programs' stress on theory tends to create a neglect of students experience of care in clinical assignments [36]. Clinical practice settings are essential in educating nursing students, and the role of visible preceptors in a permissive atmosphere is of great importance in their learning process [37]. Students need EHR training to develop competence and improved confidence in clinical settings. Integration of Academic Electronic Health Record software (AEHR) in a college setting is one way to prepare students entering the clinical workplace [38, 39].

Implementation of new practice in health care settings involves challenging changes and the use of key personnel is revealed as one important factor to ensure success [4, 40]. Implementations imply knowledge translation and require evidence-based interventions in addition to a distinct anchorage in organizational management [19, 41, 42]. The effects of educational measures on nursing documentation and nursing diagnoses point out more complete nurse plans in addition to more systematic and standardized documentation [6, 19, 43, 44].

The aim of this study is to answer the following research questions.

How do nurses experience an educational intervention of PES-structured FTF-ND and how is their clinical experience of use and usefulness?

How do nursing students assess their competence and understanding of nursing documentation in general and PES-structured FTF-ND in particular after completing their clinical practice placement in the intervention unit?

## 2. Method

### 2.1. Design and Participants

The study has a descriptive design with a qualitative approach. This is an appropriate design when the aim of a study is to describe the informants' thoughts and experiences [45]. The nurses constitute the major participant group being the main target of the intervention. The nursing students constitute a valuable perspective on use and usefulness of FTF-ND in the medical department. Their experience is therefore included to enrich the results presented in this paper.

#### 2.1.1. Nurses and Nurse Key Personnel

All nurse staff members of the actual department were given the opportunity to participate in the educational intervention (*n* = 101), with a completion rate of seventy-two (*n* = 72). Part time employment < 50% and nurses working night shift only largely explain the participation rate.

The inclusion criterion of an informant was being a nurse staff member in one of the four units constituting the medical department of the intervention. The nursing management participated in making a strategic selection of nurses, ensuring informants are representing all four units. The total sample consisted of eighteen nurses (*n* = 18). Eleven nurses were recruited as regular nurses; two of them were nurse assistants (for practical research considerations, referred to as nurses). The seven remaining participants, all nurses, were recruited by virtue of their role as* key personnel* of EHR nursing documentation. The concept of EHR-nurse key personnel defines an additional role to the ordinary nurse role. It is described as a nurse* appointed by the nurse management, who has completed specific training*,* with thorough knowledge of nurse documentation and with a special task to instruct and supervise the other nurses* (hospital's definition).

The participants' work experience as nurses ranged from 1 to 37 years, with a median of 7.5 years. Their employment percentage ranged from 75 to 100%; 14 of the 18 nurses worked in a 100% position. In 2013, the University Hospital involved in the study had 993 beds and 59016 hospitalizations. The intervention department had 41 beds.

#### 2.1.2. The Educational Intervention for Nurses and Nurse Key Personnel

A pilot study was completed prior to this paper's study [9, 46]. Results pinpointed the importance of extending the time period of the educational intervention together with discovering the advantage of centering part of the intervention to specific key persons in order to promote the implementation process. The various educational measures (Table 1) were planned, targeted, and executed in accordance with current recommendations of implementation within health research. The educational measures in our study are anchored within the concepts of* educational outreach* and* local opinion leaders*. Educational outreach is defined as “the use of a trained person meeting providers in their practice settings to give information with intent to change the providers practice” [41, p.7], [42]. The concept of local opinion leaders, being the nurse key personnel in our study, comprises various characteristics. We emphasized the features of educational influence, technical competence, and conformity to the system norms [41, 47]. The educational measures' content aimed at enhancing clinical reasoning in the diagnostic process [19]. The first and second authors of this paper acted as instructors.

#### 2.1.3. Nursing Students

The student sample consisted of second year undergraduate nursing students (*n* = 6). They were informed of the ongoing intervention study at the specific department prior to making their choice in clinical practice placement. The sample consisted of all students completing their clinical practice in the intervention department during the period of the study. Completion of practice constituted the inclusion criteria. The students did not participate in the nurses' educational intervention but were prepared theoretically and practically along with their fellow students. The content was consistent with the intervention's educational measures, although in a college setting. This was completed before entering their eight weeks of clinical practice. The first and second authors acted as instructors for the students.

### 2.2. Data Collection

The use of focus groups has increased within health research. Methodological advantages are related to group dynamics as the group members react to what is being said and in this manner lead to deeper expressions of understanding and opinion. This is a contributing factor in generating in-depth data. In addition, they are efficient in the manner of gathering a variety of viewpoints in a short time [45].

We conducted five focus group interviews upon closure of the intervention period: two nurse interviews (*n* = 5 + *n* = 6), one nurse key personnel interview (*n* = 7), and two student interviews (*n* = 3 × 2). The first and second authors conducted the interviews, one as a moderator and one as an observer who was taking notes. The interviews lasted from 30 to 55 minutes, with an average of 40 minutes.

Effective focus group interviews need a well-planned interview guide, starting from general to specific questions [45]. We developed two semistructured interview guides of main topics (Table 2), following specific questions, one for nurses/nurse key personnel and one for students.

### 2.3. Data Analysis

The data was analyzed using qualitative content analysis as described by Graneheim and Lundman [48]. Trustworthiness in qualitative content analysis depends on the systematic, thorough completion of data collection, analysis, and result reporting. It is crucial to present the results in a valid and understandable manner [49]. Principal concepts describe the steps in the analyzing process [48]. We interpreted them as follows: the transcription of interviews represented the* unit of analysis*; the domains in the interview guide formed the basis of the* content areas*. The transcripts were initially adjusted by means of a preliminary condensation, sorting out the main content but cautiously keeping the original quotations in their context. Identifying the meaning units in the text, condensation of these units, isolating codes and categories, and finally developing the themes were the following steps. Two of the authors read the transcripts separately and came to a mutual understanding in the analyzing steps.  Table 3 illustrates the analyzing process. We followed these steps rigorously through identification of* all* themes presented in this paper.

The five focus group interviews resulted in three separate analyses: one analysis including data from the two nurse groups, one based on the key personnel group, and one based on the two student groups. This was done in order to keep a thorough overview of the results before comparing them as part of a joint context. The main results are presented in Tables 4, 5, and 6.

### 2.4. Ethical Considerations

Ethical questions were cautiously considered. An inquiry was sent to the Regional Committees for Medical and Health Research Ethics in Norway. The study was assessed as a quality assessment project of the actual hospital and therefore ethically reviewed and sanctioned by the hospital's ethical protection authority. The management at the department gave further approval to the study.

The focus group participants were informed of the aim and purpose of the study both orally and in writing and signed a consent form prior to the interview. The data was anonymized, handled, and kept secure, according to regulations of research ethics.

## 3. Results

Main results are illustrated in Tables 4, 5, and 6. Quotations are included in the following text to highlight the themes diverging from the three analyses.

### 3.1. Nurses

The content areas of the analysis are presented through key features of* preparations*,* nurses at work*, and* the way ahead*.

*Preparations*. The majority of the nurses talked about the educational measures as being useful, giving them an opportunity to discuss and reflect on the use of FTF-ND. The practical sessions with authentic patient cases prepared by the nurse key personnel were described as particularly meaningful.

*“Surely, when one only gets a theoretical explanation, it all seems both advanced and comprehensive, but as soon as you get to try it yourself, it is not!”*

*Nurses at Work*. This content area represents the nurses' descriptions of clinical use. Their descriptions were associated with the concept of critical thinking and the fact that the use of FTF-ND positively affected the nursing process. Reflection, discussions, and focus on cooperation became apparent.

*“One can help one another, because one gets uncertain of how to formulate a lot of times … do other people understand … is it completely unclear? This is important in order to get the message correct!”*

On the other side, the nurses talked about challenges in writing the nursing diagnoses and finding the best expressions and emphasized the time consuming factor due to this. The nurses stressed the importance of the nurse key personnel role as a facilitator of nursing documentation. Descriptions of frustration were apparent by the nurses as well as the key personnel. These were largely related to PC shortage and working conditions.

*The Way Ahead*. The nurses focused on the importance of teamwork and support in order to keep a durable attention on nursing documentation.

### 3.2. Nurse Key Personnel

The analysis content areas are organized as key features of* preparations*,* the key personnel role, nurses at work*, and* the way ahead.*

*Preparations*. The experiences of the educational measures were related to the importance of meeting the nurse group in a well-prepared, trustworthy manner, empowered in their role as key personnel.

*“You have in a way given nurse documentation a face, in a way that they (the nurse staff) somehow know: you can help me, because this is something you know well.” *

The key personnel nurses reported the follow-up guiding period as useful. 

*The Key Personnel Role*. The nurse key personnel stated positive experiences related to their role in the study as it seemingly promoted a clarification in their role among the nursing staff. They described how the study enabled a deeper focus on the unit's documentation status in addition to a more thorough overview of the individual nurse's competence.

*“We might have uncovered the ones who were a bit insecure of it (nursing diagnoses) in the beginning, and made them more confident.”*

*Nurses at Work*. The nurse key personnel talked about how implementation of FTF-ND positively affected the steps of the nursing process, promoting reflection and patient focus. The perspective of usefulness was prominent. This quotation refers to the nursing diagnosis' PES-structure.

*“One consequence is that it has become easier, thinking like that … of the different aspect belonging together.” *

Use of FTF-ND as a factor stimulating collaboration was apparent.

*“It may stop sometimes, how to articulate and state the problem, one can need that help, more heads are better than one …”*

*The Way Ahead*. The informants talked about an optimistic and positive view of nursing diagnoses' future use along with a perception of usefulness.* “We are really getting a grip of it now, it's a good tool, I think it is here to stay”.* They expressed at the same time a concern related to resources of time and organizational adjustments needed to utilize the intensions of their specific role. The nurse key personnel described this as necessary factors enabling them to keep up the focus and motivation of nursing documentation among the nurse staff.

*“The challenge now is to keep the focus, and when new nurses start, that they get introduced to how we work.”*

### 3.3. Nursing Students

*Preparations*,* nursing student in practice*, and* the student assignment/nurse care plan* display the key features of the analysis' content areas. 

*Preparations*. The students described an understanding on how to use FTF-ND and the educational measures prior to clinical practice as useful. They expressed a wish of practical training related to the use of EHR before entering the clinical setting in order to understand the nursing documentation system.

*“… because you have to do it yourself in order to remember, there is no use sitting there, watching someone else doing it.”*

*Nursing Student in Practice*. The students pointed out a secure learning environment characterized by discussions, with involved visible preceptors. Their descriptions were dominated by perceptions of units with evident focus on nursing documentation and few instances of the opposite. The students talked about how they actively participated in patient assessments and in developing nurse care plans.

*“It has been really positive, we have been discussing how to formulate and express it in order to make it concise and there has been a lot of useful dialogue – all through the period.”*

*Student Assignment/Nurse Care Plan*. The students reported a positive learning outcome but stated at the same time mixed experiences on how to integrate the clinical and practical aspects to theory. One student said the following.

*“How on earth should I put it in order for others to understand my thoughts?” *

## 4. Discussion

The article reports results of nurses' and nursing students' experiences after implementation of FTF-ND. We wish to highlight findings related to the study's educational measures and the informants' experiences of use and usefulness.

### 4.1. The Educational Measures

We used various educational measures in order to carry out the intervention's educational outreach [41]. We emphasized the use of depersonalized patient cases in order to promote reflection and discussion on how to understand the use of FTF-ND (Table 1). The practical group session of 3–5 nurses, organized after a strategic theoretical introduction of nursing documentation in general and FTF-ND in particular, was described as particularly meaningful. It clearly related the concept of nursing diagnosis to the nurses' clinical everyday life and challenged their clinical reasoning. The results indicate how this activity seemed to generate recognition and utility value. Emphasizing real patient cases is in accordance with acknowledged research findings. Staff education measures are found to be essential in order to understand and utilize nursing diagnoses. It is recommended to focus on diagnostic reasoning in order to increase the nurses' ability of diagnostic accuracy. Guided clinical reasoning employs real patient cases, and the aim is to promote critical thinking and reflection. Effect of such guidance reveals enhanced quality in nursing documentation [6, 19, 43]. The length of guiding periods is stated as an important factor when implementing the use of nursing diagnoses [19]. Based on the findings in the study's pilot [9, 46], the guidance follow-up period was extended from a period of 9 weeks to a period of 7 months. As illustrated in Table 1, we targeted this specifically to the key personnel acting as local opinion leaders [41] within their units. We discovered that a distinct emphasis on the key personnel proved to be particularly important. The aim was to anchor the project within the unit and it contributed in giving the key personnel a thorough overview of the nursing documentation status in their unit. The investment of giving them the extra time to understand the theory and practical use of FTF-ND was strategic as it prepared them for the active role among the nursing staff. The role of local opinion leaders is to promote positive change within the peers by being a visible role model, offering professional support. Their role is significant in an implementation process seeking change of practice [40–42]. Our analysis reveals that the nurses perceived the key personnel as positive reminders, supporting them in their work and promoting consciousness of FTF-ND use. This is a result supporting essential characteristics in a local opinion leader, as we interpret it [41, 42]. The content of the guidance sessions involved focus on practical challenges of FTF-ND use, based on the key personnel's experiences within their specific unit. Depending on the individual's needs, we used the unit's care plans as a basis for the guiding sessions, emphasizing clinical reasoning in specific patient cases, and adjusted the guiding content to the expressed needs throughout the follow-up period. An additional aim in the targeted guidance was to support the key personnel in their role as local opinion leaders, facilitating use of FTF-ND among their nurse colleagues. We found that the key personnel were empowered in their role. This relates to feeling more confident in nursing documentation in addition to being more visible in their role.

The nursing students were educated in using FTF-ND by the same instructors as the nurses. Findings indicate that the students felt prepared to use FTF-ND but they expressed a wish in extended specific computerized training. Recent research supports the students in this respect as ICT (Information Communication Technology) in general and virtual EHR training specifically are considered essential to prepare them [39, 50, 51]. We discovered a positive synergy effect as the nursing students met nurses in a clinical setting speaking the “same language” as them, possessing the same theoretical understanding of FTF-ND. This finding relates to the well-known practice theory gap described in nursing. It pinpoints the importance in students experiencing an evidence-based practice, as in our study, meeting nurses actually using FTF-ND [37, 52]. We consider this finding a valuable example of a positive effect of collaboration between the educational and clinical field in order to endorse quality of practice and reduce the practice theory gap.

### 4.2. FTF-ND Use and Usefulness

The nurses described FTF-ND as useful, supporting them in their diagnostic process as it challenged them to perform a deeper analysis into the patient's health needs. This is particularly seen in how it seemingly increased their reflection in identifying the connections between the health need (P), what it relates to (E), and its clinical symptoms (S). The PES-structure seemed to function as a tool in the diagnostic process. Our findings support the association between the use of nursing diagnoses and critical thinking [25, 27, 30]. Lunney [27] describes the difficulty in identifying the best and most accurate diagnosis in order to cover the complexity of a patient's health need. This requires a continuous development in critical thinking. We saw that the use of FTF-ND challenged the nurses and nursing students as their awareness of patient and nursing needs seemed to be affected. Our findings disclose how FTF-ND caused discussions among the nurses and nursing students not only on how to find the right words describing the individual diagnosis but also on how it contributed to an increased focus in nursing documentation in general. Tables 4 and 5 show how the implementation of FTF-ND in many ways positively affected the various steps in the nursing process. The identification of FTF-ND seemed to assist them in their work; they experienced improved and more complete nurse care plans. This is in accordance with other research findings revealing positive consequences in nursing diagnosis use [13, 19, 20, 27, 53].

The nursing students' observations largely support the nurses' descriptions of FTF-ND usefulness plus the units' evident focus on nursing documentation in general. The students represent a valuable perspective as observers and participants of nursing documentation during their placement period in the intervention units. They were included and acknowledged as resourceful partners, experiencing that their views mattered (see Table 6) and described enhanced understanding of nursing documentation and the use of FTF-ND after their practice period. They got the opportunity to participate actively and establish and independently work on the nurse care plans, with preceptors creating a safe learning atmosphere; this is a crucial factor for nursing students in their clinical practice [37]. Their descriptions of the unit uncover an ability of critical thinking as they assessed the nurses' work routines, the nurses' patient focus, and commitment to nursing documentation. The student assignment which consists of a patient care plan, supplemented with a requirement of reflection over the patient's nursing needs, is emphasized due to how it relates to a student's ability of critical thinking and use of FTF-ND. The students' descriptions are in accordance with current research. The outcome seemed to affect their critical thinking ability, but the education's theoretical emphasis caused frustration [34–36].

### 4.3. Barriers of FTF-ND

Nurses described difficulties in finding the appropriate formulations of accurate nursing diagnoses covering the patient's specific health needs. Nurses need working tools to support them in their work process; and hence, this finding is an important issue to highlight. Despite their descriptions of nursing diagnoses as being useful and enhancing reflection and consciousness, the struggle of find the right words clearly occupied them. It is however interesting to see how a recent study revealed no significant difference in amount, quality, or category of nursing interventions when using NANDA classification compared to FTF-ND [31].

### 4.4. Methodological Considerations

Focus group interviews may imply challenges as some may be uncomfortable expressing their view in front of others. The dynamics of the group may generate a group culture that prevents the individual members' expressions, with a “group think” dominating the session. The moderator's role is to ensure that all voices are heard [45].

We experienced some challenges relating to these factors. A few dominating informants characterized parts of the interviews. This represented an important task for the moderator and demanded close attention. It also made us carefully assess the results. Each of the student focus groups consisted of three students. Six to ten people in a group is considered ideal size; groups of four or fewer might generate inadequate interaction [45]. The six students, completing their practice in two split periods, were the department's only students during the intervention period. They formed a homogenous group, related to their student role and clinical experience. They interacted adequately and showed a genuine interest in the themes.

Despite the challenges identified, we assessed the group dynamics as successful and we gained relevant data covering the main topics.

### 4.5. Conclusion—Clinical Implications

The educational measures of this study seemed to enhance the nurses' understanding of FTF-ND and prepare them for clinical use. Our findings support the emphasis of key personnel in an implementation process. The nurses experienced implementation of FTF-ND as a useful tool in the diagnostic process, assisting them in their work and affecting their critical thinking ability positively. Finding the most accurate formulation is at the same time a present challenge. Despite this, our findings indicate a valid clinical usability of FTF-ND. The use of standardized nursing diagnoses is partly implemented in some regions of Norway but largely in the starting line. The findings in our study support the need for thorough evidence-based planning and effort when the aim is to implement nursing diagnoses. Clinical use of FTF-ND may thereby serve as valuable preparation for standardized nursing diagnoses. The nursing students served as an indicator to the intervention units' focus on nursing documentation and largely supported the nurses' descriptions. The nursing students' descriptions indicate a positive learning outcome related to their understanding of FTF-ND use and nursing documentation in general.

## Figures and Tables

**Table 1:** Educational measures in the intervention—nurses and nurse key personnel.

Phase	Structure and content	Duration and participants	Intension
1	Teaching session: Understand the theory and use of FTF-ND, PES structure, and nursing process. Focus on nurse key personnel as local opinion leaders in the study. Group work max 2-3 persons: practical training using depersonalized patient data.	6 hours Nurse key personnel: 7 persons Unit leaders: 5 persons Instructors: 2 research team members	Prepare nurse key personnel. Ensure their understanding. Include them in the preparations of phase 2. Include nursing management as strategic personnel.

2	Teaching session: Understand the theory and use of FTF-ND, PES structure, and nursing process. Group work max 3–5 persons: practical training using depersonalized patient data.	4 hours Nurse staff members: 25–30 persons Repeated: 3 times Instructors: 2 research members, with nurse key personnel actively contributing as coinstructors.	Prepare the nurse staff members on theoretical and practical use. Create a collective experience. Ensure joint knowledge.

3	Guidance and counselling follow-up period: Practical use of FTF-ND and focus on use and challenges.	Initial 2 months: every second week Last 5 months: once a month/or based on individual needs Nurse key personnel: one-one guidance. Instructors: 2 research team members	Support the nurse key personnel in their role and offer professional support on use of FTF-ND.

4	Teaching session: Reflection and discussion of use and usefulness. How to go on after closure of study	6 hours Nurse key personnel: 7 persons Unit leaders: 5 persons Instructors: 2 research team members	Summarize experiences. Identify action after closure of study. Include nursing management as strategic personnel.

**Table 2:** Interview guide—main topics.

Main topics	Nurses/nurse key personnel	Nursing students
(1)	Experiences related to the educational measures, their understanding, usefulness, and being prepared for clinical use **Additional nurse key personnel**; experience of their role as local opinion leaders in their unit and in the study	Experiences related to the educational measures, their understanding, usefulness, and being prepared

(2)	The use of FTF-ND specifically and nursing documentation generally in their clinical work	The use of FTF-ND specifically and nursing documentation generally in their clinical work. Perceptions of the units' and preceptors' focus on FTF-ND/nursing documentation.

(3)	Thoughts and reflections of the way ahead as a unit, after closure of the intervention study **Additional nurse key personnel**; thoughts of their role after closure of study	Perceptions and experience related to the clinical assignment—nurse care plan: being prepared, execution, utility value

**Table 3:** Illustration of the analyzing process, example from text to theme.

Content area	Meaning unit	Condensation of meaning unit	Code	Category	Theme
Nurses at work using the nursing process: *Nursing diagnoses*—*FTF-ND *	*I think it's really, really useful because so much information is concentrated at once *	It's really useful, the information is concentrated	Useful to concentrate	Useful	Nursing diagnoses enhance critical thinking Focus on usefulness
	*It's easier to see the totality, really, after we started with nursing diagnoses *	Easier to see the totality with nursing diagnoses	Grasp the totality		
	*Worth the struggle? Yes, I do mean that, kind of related to being more conscious of nursing *	More conscious of nursing	Increases consciousness	Increased awareness of patient's needs requires more reflection and is time-consuming	
	*It isn't always obvious, just coming to you, so clearly, that bit requires more of the documentation than before, one has to think a bit more, and therefore use more time *	Not always obvious, requires more now, have to think more, use more time	Requires more reflection and time		

**Table 4:** Results from qualitative content analysis—nurses.

Content area	Categories	Themes
**Educational measures**: Teaching sessions, practical and theoretical	Consciousness and reflection Usefulness and recognition Challenging before group work	Educational preparations are useful

**Nurses at work**: Nursing documentation in general	Care plans: better overview, ease workload Increased focus	Increased focus on nursing documentation
**Nurses at work**: Frustrations	Challenges r.t framework: inaccessible equipment, interference, disturbance	External factors are barriers
**Nurses at work**: Nursing process *Assessment* *Nurse diagnosis FTF-ND* *Planning, goals* *Interventions* *Evaluation/outcome *	Assessment is crucial FTF-ND: Focus on patient empowerment Useful Increased awareness of patient's needs Increased reflection, but time-consuming Goals are difficult but ensure direction Evaluation gets easier Initial skepticism	Nursing diagnoses enhance critical thinking Focus on usefulness
**Nurses at work**: Collaborating on nursing documentation	Day shifts are essential Encourage, support, and help Challenging, when alone	Support and security dominate Visible, present, and supportive nurse key personnel
*The role of nurse key personnel *	Distinct support initially Visible, promotes consciousness, a reminder	

**The way ahead**: Nursing diagnoses FTF-ND, documentation in general	Help and recognize each other Organize the work Continuation of ND use	Teamwork/collaboration is essential

**Table 5:** Results from qualitative content analysis—nurse key personnel.

Content area	Categories	Themes
**Educational measures**: Teaching sessions, practical and theoretical	Well prepared as nurse key personnel Positive experience to participate in teaching	Essential to be well prepared

**The nurse key role**:	Clarification of the nurse key role Positive organizational opportunities Useful guidance Practical challenges	Empowerment of the nurse key role

**Nurses at work**: Nurse documentation, in general	Increased professional focus Mutual educational experience promotes teamwork Development of practical solutions	Increased focus on nursing documentation
**Nurses at work**: Frustrations	Challenges r.t framework: inaccessible computers interference, interruptions	External factors are barriers
**Nurses at work**: Nursing process *Nurse careplan (NCP)* *Assessment* *Nurse diagnosis FTF-ND* *Planning, goals* *Interventions* *Evaluation/outcome *	NCP: positive development structure, improvement Emphasis on patient empowerment FTF-ND: Useful Increased consciousness Goals ensure direction Clarifies interventions Evaluation gets easier	Nursing diagnoses enhance critical thinking and affect completeness of the nursing care plan Focus on usefulness
**Nurses at work**: Collaborating on nurse documentation	More discussions, common focus Importance of organizing the work	Nursing documentation—joint mission

**The way ahead**: The future nurse key role	Balance and maintaining focus on documentation, motivate Requires time and effort	Maintain a visible focus, requires resources

**Table 6:** Results from qualitative content analysis—nursing students.

Content area	Categories	Themes
**Educational measures**: Nursing documentation in school, practically and theoretically	Discover personal development Discussions and practical training are valuable Need clinical training Hope for future AEHR Feel insecure	Training prior to practice is valuable, wish for more
Learning nursing diagnoses FTF-ND in particular:	Gradually increased understanding from 1 to 2 years Reflection and training	Understand the use of nursing diagnoses

**Nursing student in practice**: The intervention unit's focus on nursing documentation	Evident focus Noticeable discussions of use and formulations of ND's Occasions of low assessment/work by routine	The nursing students use their critical thinking when observing the unit
**Nursing student in practice**: The preceptor's focus on nursing documentation	Distinct and well-planned counselling/guidance Acknowledgement Positive learning environment Occasions of vague guidance from others beside preceptors	Acknowledged and empowered in a secure learning environment
**Nursing students in practice**: Nursing process *Nurse careplan (NCP)*: *Assessment* *Nurse diagnosis FTF-ND* *planning*,* goals* *Interventions* *Evaluation/outcome *	The EHR system requires training Participation NCP: promotes independence and consciousness Data collections require training More apparent focus this year Can feel insecure on what, where, and how to formulate	Utilization promotes nursing consciousness/critical thinking. Displays challenges of utilization

**The student assignment**: Nurse care plan including a reflection/assessment component	Discover personal development Enhance consciousness Difficult to communicate the theoretical foundation of clinical practice Stressful	Positive learning outcome, challenges critical thinking. Displays a stressful factor

## References

[1] Nøst T. H., Tettum B. I., Frigstad S. A. N-catch II—et granskningsinstrument for vurdering av sykepleiedokumentasjon.

[2] Paans W., Sermeus W., Nieweg R. M. B., van der Schans C. P. (2010). D-Catch instrument: development and psychometric testing of a measurement instrument for nursing documentation in hospitals. *Journal of Advanced Nursing*.

[3] André B., Frigstad S. A., Nøst T. H., Sjøvold E. Exploring communication in stressful situations among nurses at a hospital's department of medicine—a correlation study.

[4] André B., Sjovold E., Rannestad T., Holmemo M., Ringdal G. I. (2013). Work culture among healthcare personnel in a palliative medicine unit. *Palliative and Supportive Care*.

[5] Dahl K., Skaug E. A., Kristoffersen N. J., Nortvedt F., Skaug E. A. (2011). Kliniske vurderingsprosesser og dokumentasjon i sykepleies. *Grunnleggende Sykepleie Bind 2*.

[6] Björvell C. (2011). *Sjuksköterskans Journalföring och Informationshantering. En Praktisk Handbok*.

[7] Collins S. A., Cato K., Albers D. (2013). Relationship between nursing documentation and patients' mortality. *American Journal of Critical Care*.

[8] Norwegian Health Personnel Act https://lovdata.no/.

[9] Nøst T. H., Blekken L. E., André B. (2014). Implementering av sykepleiediagnoser i fritekst. *Sykepleien Forskning*.

[10] Gjevjon E. R., Hellesø R. (2010). The quality of home care nurses' documentation in new electronic patient records. *Journal of Clinical Nursing*.

[11] Wang N., Hailey D., Yu P. (2011). Quality of nursing documentation and approaches to its evaluation: a mixed-method systematic review. *Journal of Advanced Nursing*.

[12] Blair W., Smith B. (2012). Nursing documentation: frameworks and barriers. *Contemporary Nurse*.

[13] Paans W., Nieweg R. M., van der Schans C. P., Sermeus W. (2011). What factors influence the prevalence and accuracy of nursing diagnoses documentation in clinical practice? A systematic literature review. *Journal of Clinical Nursing*.

[14] Urquhart C., Currel R., Grant M., Hardiker N. R. (2009). Nursing record systems: effects on nursing practice and healthcare outcomes. *The Cochrane Database of Systematic Reviews*.

[15] Saranto K., Kinnunen U. M., Kivekäs E. (2014). Impacts of structuring nursing records: a systematic review. *Scandinavian Journal of Caring Sciences*.

[16] Doenges M. E., Moorhouse M. F., Murr A. C. (2013). *Nursing Diagnosis Manual: Planning, Individualizing and Documenting Client Care*.

[17] Paans W., Sermeus W., Nieweg R. M. B., van der Schans C. P. (2010). Determinants of the accuracy of nursing diagnoses: influence and ready knowledge, knowledge sources, disposition toward critical thinking and reasoning skills. *Journal of Professional Nursing*.

[18] Carpenito-Moyet L. J. (2010). Teaching nursing diagnosis to increase utilization after graduation. *International Journal of Nursing Terminologies and Classifications*.

[19] Müller-Staub M. (2009). Evaluation of the implementation of nursing diagnoses, interventions, and outcomes. *International Journal of Nursing Terminologies and Classifications*.

[20] Paans W., Sermeus W., Nieweg R. M. B., van der Schans C. P. (2010). Prevalence of accurate nursing documentation in patient records. *Journal of Advanced Nursing*.

[21] Doenges M. E., Moorhouse M. F. (2003). *Application of Nursing Process and Nursing Diagnosis: An interactive text for Diagnostic Reasoning*.

[22] Carnevali D. L. (1992). *Nursing Care Planning*.

[23] Ehnfors M., Ehrenberg A., Thorell-Ekstrand I. (2000). *VIPS-Boken. Om en Forskningsbaserad Modell för Dokumentation och Omvårdnad i Patientjournalen*.

[24] Müller-Staub M., Lavin M. A., Needham I., van Achterberg T. (2006). Nursing diagnoses, interventions and outcomes—application and impact on nursing practice: systematic review. *Journal of Advanced Nursing*.

[25] Axelsson L., Björvell C., Mattiasson A.-C., Randers I. (2006). Swedish registered nurses' incentives to use nursing diagnoses in clinical practice. *Journal of Clinical Nursing*.

[26] Granum V., Opsahl G., Solvoll B. A. (2012). Hva kjennetegner kritisk tenkning? Literature review. *Sykepleien Forskning*.

[27] Lunney M. (2010). Use of critical thinking in the diagnostic process. *International Journal of Nursing Terminologies and Classifications*.

[28] Paul S. A. (2014). Assessment of critical thinking: a Delphi study. *Nurse Education Today*.

[29] Lunney M. (2003). Critical thinking and accuracy of nurses’ diagnoses. *International Journal of Nursing Terminologies and Classifications*.

[30] Paans W., Sermeus W., Nieweg R. M. B., Krijnen W. P., van der Schans C. P. (2012). Do knowledge, knowledge sources and reasoning skills affect the accuracy of nursing diagnoses? A randomised study. *BMC Nursing*.

[31] Falk J., Björvell C. Does the use of a classification for nursing diagnoses affect nursing students' choice of nursing interventions?.

[32] Morrall P., Goodman B. (2013). Critical thinking, nurse education and universities: some thoughts on current issues and implications for nursing practice. *Nurse Education Today*.

[33] Vågan A., Erichsen T., Larsen K. (2014). En mixed methods studie: Sykepleierstudenters syn på kunnskap og læring. *Sykepleien Forskning*.

[34] Marchigiano G., Eduljee N., Harvey K. (2011). Developing critical thinking skills from clinical assignments: a pilot study on nursing students' self-reported perceptions. *Journal of Nursing Management*.

[35] Andreou C., Papastavrou E., Merkouris A. (2014). Learning styles and critical thinking relationship in baccalaureate nursing education: a systematic review. *Nurse Education Today*.

[36] Solvoll B.-A., Heggen K. M. (2010). Teaching and learning care—exploring nursing students' clinical practice. *Nurse Education Today*.

[37] Jonsén E., Melender H.-L., Hilli Y. (2013). Finnish and Swedish nursing students' experiences of their first clinical practice placement—a qualitative study. *Nurse Education Today*.

[38] Baillie L., Chadwick S., Mann R., Brooke-Read M. (2013). A survey of student nurses' and midwives' experiences of learning to use electronic health record systems in practice. *Nurse Education in Practice*.

[39] Gloe D. (2010). Selecting an academic electronic health record. *Nurse Educator*.

[40] André B., Ringdal G. I., Loge J. H., Rannestad T., Kaasa S. (2008). The importance of key personnel and active management for successful implementation of computer-based technology in palliative care: results from a qualitative study. *CIN: Computers Informatics Nursing*.

[41] Grimshaw J. M., Eccles M. P., Lavis J. N., Hill S. J., Squires J. E. (2012). Knowledge translation of research findings. *Implementation Science*.

[42] O'Brien M. A., Rogers S., Jamtvedt G. (2007). Educational outreach visits: effects on professional practice and health care outcomes. *Cochrane Database of Systematic Reviews*.

[43] Bruylands M., Paans W., Hediger H., Müller-Staub M. (2013). Effects on the quality of the nursing care process through an educational program and the use of electronic nursing documentation. *International Journal of Nursing Knowledge*.

[44] Rykkje L. (2009). Implementing electronic patient record and VIPS in medical hospital wards: evaluating change in quantity and quality of nursing documentation by using the audit instrument Cat-ch-Ing. *Nursing Science*.

[45] Polit D. F., Beck C. T. (2012). *Nursing Research: Generating and Assessing Evidence for Nursing Practice*.

[46] Nøst T. H., Blekken L. E., Andrè B. Sykepleieres erfaringer med sykepleiediagnoser etter P(R)ES struktur—en pilot studie.

[47] Flodgren G., Parmelli E., Doumit G. (2011). Local opinion leaders: effects on professional practice and health care outcomes. *Cochrane Database of Systematic Reviews*.

[48] Graneheim U. H., Lundman B. (2004). Qualitative content analysis in nursing research: concepts, procedures and measures to achieve trustworthiness. *Nurse Education Today*.

[49] Elo S., Kaariainen M., Kanste O., Polkki T., Utriainen K., Kyngas H. (2014). Qualitative content analysis: a focus on trustworthiness. *SAGE Open*.

[50] Taylor L. A., Hudson K., Vazzano J., Naumann P., Neal M. (2010). The electronic health record meets baccalaureate nursing curriculum: stories from the battlefield. *Nurse Leader*.

[51] Button D., Harrington A., Belan I. (2014). E-learning & information communication technology (ICT) in nursing education: a review of the literature. *Nurse Education Today*.

[52] Hartigan I., Cummins A., O'Connell E. (2009). An evaluation of lecturer practitioners in Ireland. *International Journal of Nursing Practice*.

[53] Thoroddsen A., Ehnfors M., Ehrenberg A. (2011). Content and completeness of care plans after implementation of standardized nursing terminologies and computerized records. *Computers Informatics Nursing*.

